# A single-arm phase II trial of *UGT1A1* genotype–guided high-dose irinotecan rechallenge in refractory metastatic colorectal cancer

**DOI:** 10.3389/fonc.2026.1907944

**Published:** 2026-07-22

**Authors:** Hana Kim, Joohyun Hong, Sun-Young Kong, Moon Ki Choi

**Affiliations:** 1 Center for Colorectal Cancer, Research Institute and Hospital, National Cancer Center 323, Goyang, Republic of Korea; 2 Department of Laboratory Medicine, Research Institute and Hospital, National Cancer Center 323, Goyang, Republic of Korea

**Keywords:** irinotecan rechallenge, later-line cancer treatment, metastatic colorectal cancer, phase II, precision medicine, UGT1A1 genotype

## Abstract

**Background:**

There is an unmet need for optimal third- or later-line treatment options for refractory or metastatic colorectal cancer (mCRC) patients. This phase II study investigated whether high-dose irinotecan rechallenge based on *UGT1A1* genotype improves the 12-week disease control rate (12w DCR), objective response rate (ORR), progression-free survival (PFS), overall survival (OS), and safety in refractory mCRC patients.

**Methods:**

Patients who had previously received more than two lines of chemotherapy, including 5-fluorouracil, oxaliplatin, irinotecan and had shown either a partial response or durable response for more than 24 weeks to irinotecan were included. Patients without the defective allele of *UGT1A1* (*UGT1A1* *1/*1) and one defective allele (*UGT1A1* *1/*6, *1/*28) were treated with intravenous irinotecan at doses of 300 mg/m^2^ and 250 mg/m^2^, respectively, every 2 weeks until disease progression or unacceptable toxicity.

**Results:**

A total of 32 patients was enrolled between October 2020 and March 2023. The primary endpoint, 12w DCR was 40.6% (13 of 32 patients). The ORR was 15.6% (5 of 32). The median OS was 9.3 months (95% CI, 5.3 to 13.3) and the median PFS was 2.9 months (95% CI, 2.5 to 3.3). Grade 3 or higher adverse events were observed in 19 patients (59.4%). Dose reduction due to adverse events occurred in 9 patients (50.0%) in *UGT1A1* wild-type group and 4 patients (28.6%) in the heterozygous group.

**Conclusion:**

High-dose irinotecan rechallenge guided by UGT1A1 genotype was feasible and showed meaningful disease control in third-line or later settings for mCRC patients previously responsive to irinotecan.

## Introduction

Colorectal cancer (CRC) is the second leading cause of cancer-related death worldwide (1) and the 5-year survival rate for metastatic colorectal cancer (mCRC) remains low (~14%) (2). First- and second-line treatment options for mCRC typically include fluoropyrimidine-based regimens combined with irinotecan or oxaliplatin, along with targeted agents such as anti-vascular endothelial growth factor (anti-VEGF) agent, bevacizumab, and the anti-epidermal growth factor receptor (anti-EGFR) agent, cetuximab, particularly in patients with RAS wild type tumors, which have been shown to improve survival outcomes (3). Regorafenib or trifluridine/tipiracil hydrochloride (TAS-102) have been approved as third-line or later treatment options, but their efficacy remains unsatisfactory, and there are concerns about significant treatment-related adverse events (4–9). In a study comparing TAS-102 plus bevacizumab versus TAS-102 alone, the combination group demonstrated a median progression-free survival (PFS) of 5.6 months and a 12-month overall survival (OS) rate of 43%, while the TAS-102 alone group showed a median PFS of 2.4 months and a 12-month OS rate of 30% (10). Another study comparing fruquintinib with placebo in metastatic CRC reported that fruquintinib extended overall survival by 2.6 months compared with placebo (11). Although both studies showed improved PFS and survival outcomes compared to their respective control groups, the observed benefits were clinically modest. The optimal treatment for refractory mCRC remains uncertain. Consequently, there is an unmet need for more effective and safer third- or later-line treatment options for refractory or mCRC patients.

The most widely used chemotherapeutic agent for mCRC is 5-fluorouracil (5-FU), a synthetic fluorinated pyrimidine analogue of uracil. It is commonly administered as part of combination regimens such as FOLFOX and FOLFIRI, which include oxaliplatin or irinotecan, respectively, and are often used in conjunction with bevacizumab as frontline treatments for mCRC (12, 13). However, most patients eventually develop resistance to standard chemotherapy and experience disease progression. In addition, 5-FU therapy can be predictably toxic in patients carrying polymorphisms in the dihydropyrimidine dehydrogenase (*DPYD*) gene, which impair hepatic degradation of the drug (14). Consequently, personalized treatment strategies based on pharmacogenetic biomarkers have become increasingly important for optimizing treatment efficacy and minimizing toxicity.

Irinotecan is a topoisomerase inhibitor and is most frequently used in combination with other drugs to treat advanced or mCRC. Common side effects of irinotecan include diarrhea, vomiting, hair loss, shortness of breath, fever, and marrow suppression (15). In the body, irinotecan is converted into an active metabolite known as SN-38, which is then detoxified by a uridine diphosphate (UDP) glucosyltransferase (UGT) enzyme encoded by the *UGT1A1* gene. Consequently, the risk of irinotecan toxicity increases with genetic variants associated with UGT enzyme activity, such as UGT1A1*28 (16). Several studies have shown that the UGT1A1*28 allele is related to irinotecan-induced adverse events (17, 18). Patients who possess two copies of the UGT1A1*28 allele (homozygous, *UGT1A1* *28/*28) are more likely to develop neutropenia following irinotecan treatment (19).

Because current third-line and later treatment options have demonstrated limited therapeutic efficacy, several recent studies have compared chemotherapy rechallenge with agents such as regorafenib, TAS-102, or fruquinitinib (9, 20, 21). Irinotecan rechallenge with or without cetuximab has also been reported in the literature several times (9, 22, 23). A retrospective study of 64 patients treated with irinotecan retreatment reported a median PFS of 5.5 months, an ORR of 23.5%, and a DCR of 78.2%, suggesting that irinotecan retreatment may remain an effective therapeutic option in carefully selected patients (23). Furthermore, a large retrospective propensity score analysis comparing chemotherapy rechallenge/reintroduction with regorafenib or trifluridine/tipiracil demonstrated superior clinical outcomes with chemotherapy rechallenge/reintroduction, including longer PFS (6.1 vs. 3.5 months; 5.5 vs. 3.9 months after propensity score adjustment), higher ORR (28.6% vs. 1.4%), and higher DCR (57.9% vs. 19.2%) (9). These findings support the potential value of chemotherapy rechallenge strategies in the later-line treatment of metastatic colorectal cancer. However, prospective data evaluating genotype-guided irinotecan rechallenge are lacking, and the optimal dosing strategy according to *UGT1A1* genotype remains undefined. Therefore, we investigated the efficacy and safety of high-dose irinotecan rechallenge based on *UGT1A1* genotype in refractory mCRC as a later-line option in this prospective study. The use of high-dose irinotecan carries the potential risk of unintended bone marrow suppression. To prevent treatment-related adverse events, especially neutropenia, we planned to perform *UGT1A1* genotyping before initiating irinotecan administration.

## Methods

### Trial design and patients

**Figure 1:**
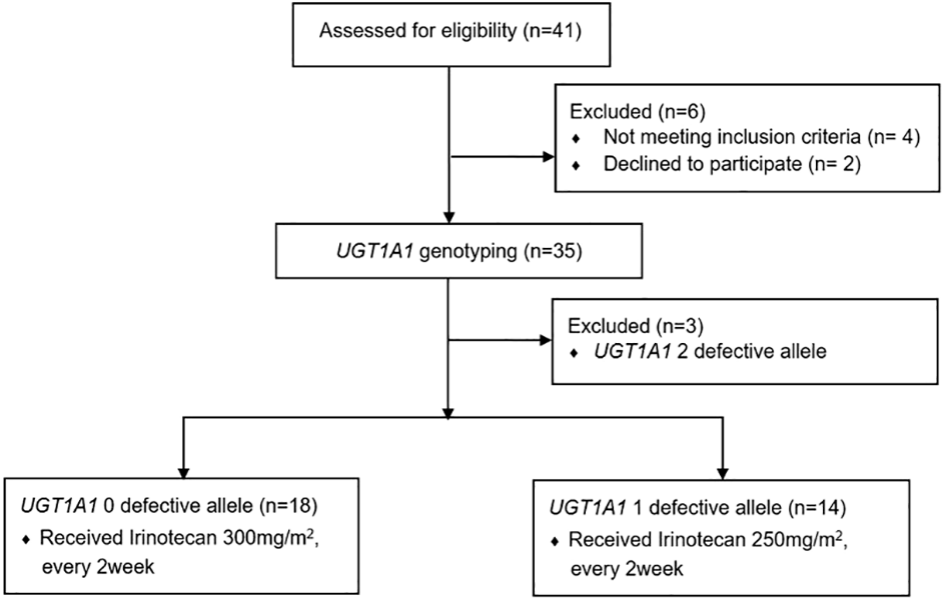
Trial profile.

The study was designed as a single-arm, prospective phase II trial. The protocol was approved by the Institutional Review Board of the National Cancer Center (IRB No. NCC2020-0100). The study was registered as the UHD trial and its approval date was March 31, 2020 (KCT0005303). Between October 2020 and March 2023, a total of 32 patients with metastatic colorectal cancer were enrolled at the National Cancer Center. Eligible patients were aged ≥19 years and had an Eastern Cooperative Oncology Group (ECOG) performance status 0 or 1. Patients were required to have received at least two prior lines of systemic chemotherapy regimens, including irinotecan-based therapy. Additional inclusion criteria included having achieved a complete response (CR), partial response (PR), or stable disease (SD) lasting at least 24 weeks during prior irinotecan-containing chemotherapy. *UGT1A1* testing was performed before administering the study drug; patients with 2 copies of the defective allele (*UGT1A1* *28/*28, *6/*6, *6/*28) were excluded. Patients without a defective allele (*UGT1A1* *1/*1) and those with one defective allele (*UGT1A1* *1/*6, *1/*28) were treated with intravenous irinotecan at a dose of 300 mg/m^2^ and 250 mg/m^2^, respectively, every 2 weeks until disease progression or unacceptable toxicity (**Figure 1**). The specific trial protocol is available in the **Supplementary Materials**.

### *UGT1A1* genotyping

Genomic DNA was extracted from peripheral blood leukocytes using Chemagic 360-D according to manufacturer’s instructions (PerkinElmer Chemagen Technologie GmbH, Germany). Conditions for PCR were as follows: denaturation for 5 min at 94 °C, 30 sec at 94 °C, 40 sec at 60 °C, and 1 min at 72 °C for 35 cycles and extension for 5 min at 72 °C with SimpliAmp (Applied Biosystems, USA). Direct sequencing was performed with BigDye^®^ Terminator v3.1 Cycle Sequencing Kit and 3730 DNA analyzer (Applied Biosystems, USA). The sequences were compared with a *UGT1A1* reference sequence (accession number: NM_000463.2) and the haplotype was annotated according to *UGT1A1* allele nomenclature guidelines (24).

### Outcomes and statistical analysis

Cancer responses were evaluated by computed tomography (CT) every 6 weeks from the initiation of chemotherapy, according to Response Evaluation Criteria in Solid Tumors (RECIST) version 1.1. Adverse events (AEs) were assessed prior to treatment, at each study visit, and for at least 100 days following treatment discontinuation. AEs were categorized and graded according to the National Cancer Institute Common Terminology Criteria for Adverse Events (NCI-CTCAE), version 4.0.

The primary endpoint was the 12-week disease control rate (12w DCR). The secondary endpoints included objective response rate (ORR), progression-free survival (PFS), overall survival (OS), and safety. The DCR was defined as the proportion of patients achieving complete response (CR), partial response (PR), or stable disease (SD). The ORR was defined as the proportion of patients achieving CR or PR. PFS was measured from the initiation date of study drug to the first observation of disease progression or death from any cause. OS was calculated from the date of chemotherapy initiation to death from any cause.

The sample size was calculated to reject 20% DCR in favor of a target DCR of 40%, with a significance level of 0.05 and power of 80%, using Simon’s two-stage design. In the first stage, 18 patients were enrolled. If 5 or more of these patients demonstrated disease control at 12 weeks, the study proceeded to the second stage. In the second stage, at least 14 additional patients were enrolled. Categorical variables are presented as counts and percentages, and continuous variables are summarized using medians and ranges. The response rates and patients’ clinical characteristics were evaluated using the χ² test or Fisher’s exact test for categorical variables. Kaplan-Meier analysis was used to estimate PFS and OS. Logistic regression analyses were performed to identify factors associated with higher likelihood of clinical benefit. All statistical analyses were performed using SPSS version 27.0.1 (SPSS Inc., Chicago, IL, USA).

## Results

### Patient characteristics

The patient demographics are presented in **Table 1**. The median age was 61 years, and 63% of the patients were male. Eighteen patients had no defective *UGT1A1* allele (UGT1A1*1/*1), while 14 patients carried one defective allele (*UGT1A1* *1/*6, *1/*28). All patients had metastatic colorectal cancer and a history of at least two systemic chemotherapy regimens; seven additional patients had received four or more lines of chemotherapy. Most tumors were located on the left side of the colon, with only one case of a right-sided tumor. The most common sites of metastasis were the lung (75.0%), liver (59.4%) and peritoneum (21.9%). The best response to prior irinotecan-based chemotherapy was partial response (PR) in 16 patients (50%) and stable disease (SD) in 16 patients (50%). The median irinotecan-free interval was 11.3 months (range 3.1 – 65.4). The *UGT1A1* gene variants and allele type of all patients are presented in **Supplementary Table 1**.

**Table 1:** Patient characteristics.

Characteristics		N = 32 (%)	
Age (years)	Median (range)	61	(47-73)
Sex	Male	20	(62.5)
	Female	12	(37.5)
ECOG performance status	0	6	(18.8)
	1	26	(81.3)
Location of primary tumor	Right-sided colon	1	(3.1)
	Left-sided colon	14	(43.8)
	Rectum	17	(53.1)
*UGT1A1* genotype	0 defective allele	18	(56.3)
	1 defective allele	14	(43.8)
Timing of metastases	Synchronous	24	(75.0)
	Metachronous	8	(25.0)
No. of metastatic sites	1	9	(28.1)
	2	11	(34.4)
	≥3	12	(37.5)
Metastatic sites	Lung	24	(75.0)
	Liver	19	(59.4)
	Peritoneum	7	(21.9)
*BRAF*, *KRAS*, and *NRAS* mutation status	All wild type	15	(46.9)
	*BRAF* mutant	0	(0)
	*KRAS* or *NRAS* mutant	17	(53.1)
No. of prior regimens	2	12	(37.5)
	3	13	(40.6)
	≥4	7	(21.9)
Prior systemic anticancer agents	Fluoropyrimidine	32	(100.0)
	Oxaliplatin	32	(100.0)
	Bevacizumab	31	(96.9)
	Cetuximab	13	(40.6)
	TAS-102	7	(21.9)
Prior irinotecan-based treatment			
Treatment line	First	14	(43.8)
	Second	16	(50.0)
	Third	2	(6.3)
Best response	Partial response	16	(50.0)
	Stable disease	16	(50.0)
Progression-free survival	Median (95% CI)	13.3	(11.8–14.8)
Irinotecan-free interval (months)	Median (range)	11.3	(3.1–65.4)

ECOG, Eastern Cooperative Oncology Group; UGT1A1, UDP-glucosyltransferase 1 family peptide A1; KRAS, Kirsten rat sarcoma; NRAS, neuroblastoma rat sarcoma.

### Efficacy

**Figure 2:**
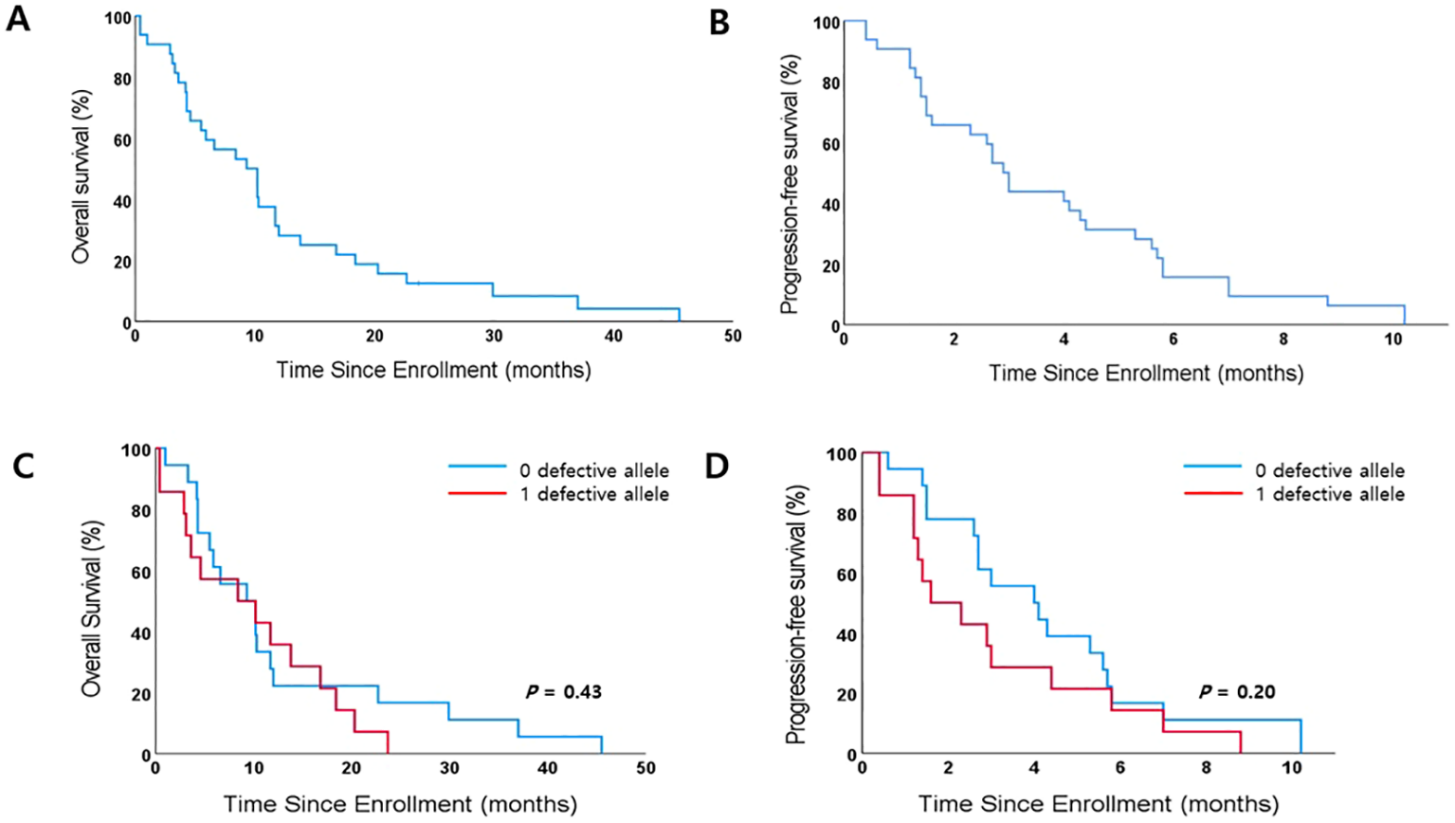
Overall survival (OS), progression-free survival (PFS). **(A)** OS, all patients group (n=32), **(B)** PFS, all patients group (n=32), **(C)** OS analysis by *UGT1A1*-defective allele status, **(D)** PFS by *UGT1A1*-defective allele status.

The data cut-off date was March 31, 2025, with a median follow-up duration of 10.0 months (range, 0.4–39.6). The primary endpoint, 12-week disease control rate (12w DCR), was 40.6% (13 of 32) including 3 partial responses (PR) and 10 stable disease (SD) responses. Based on the best overall response, the disease control rate was 65.6% (21 of 32) (**Table 2**). The objective response rate (ORR) was 15.6% (5 of 32). The median progression-free survival (PFS) was 2.9 months (95% CI, 2.5–3.3), and the median overall survival (OS) was 9.3 months (95% CI, 5.3–13.3) (**Figures 2A, B**).

**Table 2:** Treatment outcome.

Tumor response	*N* = 32 (%)	
Best overall response		
Complete response	0	(0)
Partial response	5	(15.6)
Stable disease	16	(50.0)
Progressive disease	8	(25.0)
Unable to determine*	3	(9.4)
Total disease control No. (%)	21	(65.6)
12-week DCR No. (%)	13	(40.6)
Median PFS months (95% CI)	2.9	(2.5–3.3)
Median OS, months (95% CI)	9.3	(5.3-13.3)

DCR, disease control rate; PFS, progression-free survival; OS, overall survival, * No disease evaluation results; 1 death, 2 EOTs (end of trial).

In the subgroup analysis, the median PFS was 4.0 months (95% CI, 1.7–6.3) for the *UGT1A1–*0 defective allele group and 1.6 months (95% CI, 0-3.3) for the *UGT1A1–*1 defective allele group (p=0.20). The median OS was 9.3 months (95% CI, 4.3-14.3) for the *UGT1A1–*0 defective group and 8.4 months (95% CI, 0-18.6) for the *UGT1A1*, 1 defective group (**Figures 2C, D**). Among the 14 patients with one defective *UGT1A1* allele, 8 had the *1/*6 genotype, and 6 had *1/*28 genotype. In the subgroup analysis of the *UGT1A1* *1 allele-deficient group, the OS for the *1/*6 *UGT1A1* group was 4.6 months (95% CI, 0-11.9) and the PFS was 1.3 months (95% CI, 1.0-1.6). For the *1/*28 *UGT1A1* group, the OS was 10.2 months (95% CI, 0.5-19.9), and the PFS was 2.3 months (95% CI, 0.7-3.3) (**Supplementary Figure 2**). There was no statistically significant difference in OS or PFS among the *1/*1, *1/*6, and *1/*28 genotype groups. Analyses of predictive factors for PFS, OS, and response rate are provided in **Supplementary Materials** (**Supplementary Tables S2**-**S4**). An exploratory subgroup analysis according to prior TAS-102 exposure showed that the 12-week disease control rate was 14.3% (1/7) in patients previously treated with TAS-102 and 48.0% (12/25) in TAS-102-naïve patients. ECOG PS of 1 was numerically associated with worse OS than ECOG PS 0, although the difference was not statistically significant (HR 2.699, 95% CI 0.910-8.002, p=0.073). No statistically significant factors affecting treatment outcomes were identified.

### Safety

The treatment-related adverse events (AEs) are summarized in **Table 3**. Treatment-related AEs of any grade were observed in 30 patients (30 of 32, 93.8%). The most frequently reported treatment-related AEs of any grade were nausea (68.8%), alopecia (62.5%), and mucositis (37.5%) (**Table 3**). Grade 3 or higher adverse events occurred in 19 (59.4%) patients. There was one case of treatment-related death due to sepsis. Grade 3 or 4 AEs included neutropenia (n=9), nausea (n=5), anemia (n=2), vomiting (n=2), anorexia (n=2), and diarrhea (n=1). Dose reduction due to AEs was required in 9 of 18 patients (50.0%) in the *UGT1A1* wild type group and in 4 of 14 patients (28.6%) with a single defective allele.

**Table 3:** Treatment-related adverse events.

Adverse event	*N* = 32			
	Any grade	(%)	≥Grade 3	(%)
Hematologic				
Neutropenia	11	(34.4)	9	(28.2)
Anemia	2	(6.3)	2	(6.3)
Thrombocytopenia	1	(3.1)	1	(3.1)
Non-hematologic				
Nausea	22	(68.8)	5	(15.6)
Vomiting	10	(31.3)	2	(6.3)
Anorexia	8	(25.0)	2	(6.3)
Diarrhea	8	(25.0)	1	(3.1)
Sepsis	1	(3.1)	1	(3.1)
Fatigue	11	(34.4)	0	(0)
Alopecia	20	(62.5)	0	(0)
Mucositis	12	(37.5)	0	(0)
Abdominal pain	6	(18.8)	0	(0)
Weight loss	1	(3.1)	0	(0)
Allergic rhinitis	2	(6.3)	0	(0)
Skin rash	1	(3.1)	0	(0)
Pruritus	1	(3.1)	0	(0)

## Discussion

Patients with metastatic CRC who fail second-line chemotherapy generally tend to have poor survival outcomes. Regorafenib and TAS-102 have been approved as treatment options in the later lines for patients whose disease progressed after fluoropyrimidine, oxaliplatin, and irinotecan-based chemotherapy, combined with anti-VEGF therapy and, if RAS wild type, anti-EGFR therapy. In a phase 3 trial, regorafenib, an oral multi-kinase inhibitor, extended median overall survival (OS) by 1.4 months compared to placebo (6.4 versus 5.0 months; p=0.0052). Grade 3 or 4 treatment-related AEs occurred in 54% of patients, and serious adverse events were reported in 44% (4). TAS-102 demonstrated a median OS benefit of 1.9 months over placebo (7.1 versus 5.3 months; p<0.001). Grade 3 or higher AEs occurred in 69% of the TAS-102 group and 52% of the placebo group (7). Both regorafenib and TAS-102 are viable treatment options for refractory, metastatic CRC; however, their efficacies are modest, and the treatment-related adverse events can be significant. A study comparing TAS-102 plus bevacizumab to TAS-102 alone found that the combination improved median PFS (5.6 vs. 2.4 months) and 12-month OS (43% vs. 30%) (10). The FRESCO-2 study showed that fruquintinib extended overall survival by 2.6 months over placebo in metastatic CRC (11). Although these recent treatments have demonstrated improved outcomes, they may be challenging to implement in low-resource settings. The efficacy and safety outcomes of the present study in comparison with those reported in the pivotal later-line clinical trials are summarized in **Supplementary Table 5**. Although cross-trial comparisons should be interpreted with caution because of differences in study design, patient populations, and eligibility criteria, our genotype-guided high-dose irinotecan strategy demonstrated efficacy outcomes that appear comparable to currently available later-line treatment options, while maintaining a manageable safety profile.

In this phase II study, high-dose irinotecan re-challenge resulted in a 12-week disease control rate (12w DCR) of 40.6% and an objective response rate (ORR) of 15.6%, with a manageable safety profile in patients with metastatic colorectal cancer (mCRC). The median progression-free survival (PFS) was 4.0 months in the *UGT1A1–*0 defective allele group and 1.6 months in the *UGT1A1–*1 defective allele group. The *UGT1A1–*0 defective allele group, which received high-dose irinotecan (300 mg/m²), showed a numerically longer PFS than the *UGT1A1–*1 defective allele group, which received 250 mg/m^2^ of irinotecan; however, the difference was not statistically significant (**Figure 2**). Because this study was not designed or powered to detect survival differences between genotype-defined subgroups, these findings should be regarded as exploratory and hypothesis-generating rather than evidence of superior efficacy. Confirmation in larger, adequately powered prospective studies is warranted. In addition, more detailed subgroup analyses could not be performed due to the limited number of patients. With respect to adverse events (AEs), this study employed high-dose irinotecan, and grade ≥3 AEs occurred in 59.4% of patients. In the SUNLIGHT trial, the incidence of grade ≥3 AEs was 72.4% in the TAS-102 plus bevacizumab group and 69.5% in the TAS-102 monotherapy group (10). In the CORRECT trial, regorafenib was associated with grade ≥3 AEs in 54% of patients (4). Thus, the incidence of severe AEs in our study was lower than that observed with TAS-102 but higher than with regorafenib. No new AEs were identified; the observed toxicities were consistent with the well-known adverse profile of irinotecan, including nausea, vomiting, diarrhea, and bone marrow suppression. The use of high-dose irinotecan may necessitate more proactive administration of antiemetics and antidiarrheal agents, as well as greater reliance on granulocyte-colony stimulating factor (G-CSF) in cases of neutropenia. Furthermore, *UGT1A1* genotyping prior to initiating high-dose irinotecan can be critical, as it may help guide alternative treatment strategies for patients at high risk of neutropenia or support systematic dose reduction when neutropenia occurs. Despite the *UGT1A1* genotype–guided approach, the toxicity associated with high-dose irinotecan rechallenge in mCRC was significant, and one treatment-related death due to sepsis occurred. However, the incidence of adverse events would likely have been considerably higher if patients carrying two defective alleles (*UGT1A1* *28/*28, *6/*6, or *6/*28) had not been excluded prior to study initiation. All patients had heavily pre-treated disease, and even 7 of the 32 patients had a history of TAS-102 treatment. Considering these findings, *UGT1A1* genotype-guided high-dose irinotecan may be a treatment option for selected patients with mCRC.

Among the 18 patients in *UGT1A1–*0 defective allele group who received 300 mg/m^2^ of irinotecan, 9 patients (50.0%) underwent dose reductions. Six patients had one dose reduction from 300 mg/m^2^ to 250 mg/m^2^, and the other remaining three patients had a further dose reduction from 250 mg/m^2^ to 200 mg/m^2^ due to treatment-related adverse events. In *UGT1A1–*1 defective allele group, which received an initial dose of 250 mg/m^2^, a total of 4 patients (28.6%) required dose reductions. Three of these patients had one reduction from 250 mg/m^2^ to 200 mg/m^2^, and one patient required an additional dose reduction from 200 mg/m^2^ to 150 mg/m^2^. The primary reasons for dose reductions were neutropenia and nausea and vomiting. In the *UGT1A1–*0 defective allele group, the causes of dose reduction were nausea and vomiting (4 of 9), neutropenia (4 of 9) and diarrhea (1 of 9). In the *UGT1A1–*1 defective allele group, all causes of dose reduction were due to neutropenia (4 of 4). The observed incidence of neutropenia was not higher in the *UGT1A1–*0 defective allele group, despite receiving a higher dose than the *UGT1A1–*1 defective allele group, which may be attributable to dose adjustment based on the *UGT1* genotype. Among patients who received 300 mg/m² irinotecan, dose reduction due to nausea and/or vomiting was required in four patients. Therefore, adequate antiemetic prophylaxis and premedication should be considered when administering high-dose irinotecan.

To date, over 135 genetic variants of *UGT1A1* have been identified, with the most common variant allele being UGT1A1*28 (25, 26). Pyrosequencing has been reported to be a rapid and reliable method for detecting *UGT1A1* polymorphisms (27). After genotyping for UGT1A1*28, patients homozygous for UGT1A1*28/*28 should be carefully considered before initiating irinotecan therapy, as they have an increased risk of developing severe neutropenia following treatment initiation (28). Patients with two defective *UGT1A1* alleles are generally recommended to receive a reduced dose of irinotecan (29). However, there is insufficient evidence to suggest that patients with no defective alleles are more tolerant of high-dose irinotecan than those with a single defective allele. Our study was designed with reference to a phase I trial in which the *UGT1A1* genotype–guided maximum tolerated dose was confirmed. In that study, the recommended irinotecan dose was 300 mg/m² for patients with no defective alleles and 270 mg/m² for those with one defective allele (18). However, despite this rationale, dose reduction was required in 50.0% of patients who started at 300 mg/m² in the present study. This finding raises the possibility that 300 mg/m² may have been too high as a uniform starting dose for a substantial proportion of heavily pretreated patients, although the present study was not designed to determine the optimal starting dose and did not include a lower-dose comparator. Therefore, it remains uncertain whether initiating treatment at a lower dose or adopting a stepwise dose-escalation strategy could have achieved a better balance between efficacy and toxicity. Nevertheless, dose modification is an integral part of irinotecan treatment and enables toxicity to be managed appropriately. Therefore, careful monitoring and timely dose adjustment are essential when administering high-dose irinotecan. Further studies are warranted to determine whether a true difference in irinotecan tolerance exists between the 1 defective and 0 defective allele groups.

Irinotecan is a widely used anticancer agent, and high-dose irinotecan therapy is readily accessible. It can be administered even after treatment failure with TAS-102 plus bevacizumab or with fruquintinib in patients with relatively good performance status. When guided by genotype-based patient selection prior to administration, this strategy represents a viable and feasible option, particularly in resource-limited settings. Additionally, compared to oxaliplatin, irinotecan has an advantage regarding chemotherapy-induced peripheral neuropathy, as oxaliplatin poses an irreversible, cumulative neurotoxicity that limits its rechallenge potential.

This study has several limitations. First, it was conducted as a single-arm trial with a relatively small patient population. The absence of a control group makes it difficult to determine whether the observed effects are attributable to the intervention itself or to other factors. In addition, the sample size was determined to evaluate the primary endpoint (12-week disease control rate) using Simon’s two-stage design and was not powered to detect differences in progression-free survival or overall survival between *UGT1A1* genotype-defined subgroups. Therefore, the subgroup survival analyses should be considered exploratory, and the observed numerical differences between genotype groups should be interpreted with caution, as they may reflect random variation rather than true treatment effects. Larger prospective studies with adequate statistical power are needed to validate these findings. Although direct comparisons with other third-line or later-line chemotherapies are not appropriate, real-world data from Asian patients report median OS of 7.5 months with TAS-102 and 6.5 months with regorafenib (30). In addition, another real-world study has shown a median PFS of 2.1 months for TAS-102 in Asian populations (31). An exploratory subgroup analysis showed a lower 12-week disease control rate in patients previously treated with TAS-102 than in TAS-102-naïve patients. Although this observation was based on only seven patients and should therefore be interpreted with caution, it suggests that prior TAS-102 exposure may represent an important efficacy-related confounding or prognostic factor. Future prospective studies should account for prior exposure to later-line therapies when evaluating the efficacy of irinotecan rechallenge. Additionally, our study included patients with relatively good general condition (ECOG 0 or 1) who could tolerate high doses of irinotecan. Therefore, high-dose irinotecan may not be suitable for patients with poor performance status in real-world clinical practice.

To the best of our knowledge, this is the first prospective clinical trial evaluating irinotecan retreatment based on *UGT1A1* genotype. *UGT1A1* genotyping prior to high-dose irinotecan rechallenge may provide an additional treatment option for heavily pretreated patients with mCRC in the third-line or later-line setting. Our findings suggest that a genotype-guided approach to irinotecan dosing may be feasible in selected patients.

## Conclusion

Our study demonstrated that high-dose irinotecan rechallenge guided by *UGT1A1* testing showed meaningful disease control in this patient population. This strategy may serve as a potential treatment option for later-line therapy, particularly in patients with relatively good performance status. Further randomized clinical trials are warranted to validate these findings.

## Data Availability

The original contributions presented in the study are included in the article/**Supplementary Material**. Further inquiries can be directed to the corresponding author.
