# Supplementary material for: A single-arm phase II trial of UGT1A1 genotype–guided high-dose irinotecan rechallenge in refractory metastatic colorectal cancer
Source: Front Oncol. 2026 Jul 22;16:1907944. doi: 10.3389/fonc.2026.1907944 (PMC13437338; doi:10.3389/fonc.2026.1907944)
Supplement: Supplementary file 1 [file DataSheet1.docx]

**Table S1** *UGT1A1* variants and allele type, all patients

| Subject No. | *UGT1A1* deficient allele | *UGT1A1* gene variant report | Identified variants | *UGT1A1* allele |
| --- | --- | --- | --- | --- |
| 1 | 0 | Not detected |  | *1/*1 |
| 2* | 0 | Not detected |  | *1/*1 |
| 3 | 1 | Detected | c, -3279T>G,  c, -3156G>A, (VUS)  -41_-40dupTA | *1/*28 |
| 4 | 1 | Detected | c, -3279T>G,  c, -64G>C, (VUS)  c, 211G>A | *1/*6 |
| 5* | 0 | Detected | c, -3279T>G | *1/*1 |
| 6 | 0 | Not detected |  | *1/*1 |
| 7* | 1 | Detected | c, -3279T>G,  c, -3156G>A, (VUS)  -41_-40dupTA | *1/*28 |
| 8 | 0 | Not detected |  | *1/*1 |
| 9 | 0 | Not detected |  | *1/*1 |
| 10 | 0 | Not detected |  | *1/*1 |
| 11 | 0 | Not detected |  | *1/*1 |
| 12 | 1 | Detected | c, 211G>A | *1/*6 |
| 13* | 2 | Detected | c, -3279T>G,  c, -3156G>A, (VUS)  -41_-40dupTA  c, 211G>A  c, 335C>G (VUS) | *6/*28 |
| 14 | 1 | Detected | c, 211G>A  c, 783G>A | *1/*6 |
| 15 | 1 | Detected | c, -3279T>G  c, 211G>A | *1/*6 |
| 16* | 2 | Detected | c, -3279T>G,  c, -3156G>A, (VUS)  -41_-40dupTA  c, 211G>A | *6/*28 |
| 17* | 1 | Detected | c, -3279T>G,  c, 211G>A | *1/*6 |
| 18 | 0 | Detected | c, -3279T>G | *1/*1 |
| 19 | 1 | Detected | c, 211G>A  c,996+15T>C (VUS) | *1/*6 |
| 20 | 0 | Detected | c, -3279T>G | *1/*1 |
| 21 | 1 | Detected | c, -3279T>G,  c, -3156G>A, (VUS)  -41_-40dupTA  c,996+15T>C (VUS) | *1/*28 |
| 22 | 1 | Detected | c, -3279T>G,  c, -3156G>A, (VUS)  -41_-40dupTA  c,996+15T>C (VUS) | *1/*28 |
| 23 | 0 | Detected | c, -3279T>G,  c, -3177C>G, (VUS)  c, -3175A>G (VUS)  c, -64G>C | *1/*1 |
| 24 | 0 | Not detected | c, -3345delC (VUS) | *1/*1 |
| 25* | 0 | Not detected |  | *1/*1 |
| 26 | 1 | Detected | c, -3279T>G,  c, -3156G>A (VUS)  c, -3177C>G, (VUS)  c, -3175A>G (VUS)  c, -64G>C  -41_-40dupTA | *1/*28 |
| 27 | 0 | Detected | c, -3279T>G, | *1/*1 |
| 28 | 1 | Detected | c, -3279T>G,  c, 211G>A | *1/*6 |
| 29 | 0 | Not detected | c, 996+15T>C (VUS) | *1/*1 |
| 30 | 0 | Not detected |  | *1/*1 |
| 31 | 0 | Detected | c, -3279T>G,  c, -3345delC (VUS)  c, 64G>C | *1/*1 |
| 32 | 1 | Detected | c, 211G>A | *1/*6 |
| 33 | 0 | Detected | c, -3279T>G,  c, 996+15T>C (VUS) | *1/*1 |
| 34 | 0 | Not detected |  | *1/*1 |
| 35 | 0 | Not detected | c, -3345delC (VUS) | *1/*1 |
| 36* | 1 | Detected | c, -3279T>G,  c, -3156G>A (VUS)  -41_-40dupTA | *1/*28 |
| 37* | 2 | Detected | c, -3279T>G,  c, -3156G>A (VUS)  -41_-40dupTA  c, 211G>A | *6/*28 |
| 38 | 1 | Detected | c, -3279T>G,  c, -3156G>A (VUS)  -41_-40dupTA  c, 686C>A | *1/*28 |
| 39 | 0 | Detected | c, -3279T>G | *1/*1 |
| 40 | 1 | Detected | c, -3279T>G,  c, -3156G>A (VUS)  -41_-40dupTA | *1/*28 |
| 41 | 1 | Detected | c, 211G>A  c, 996+15T>C (VUS) | *1/*6 |

*: Screening-failed patients; VUS, variant of uncertain significance

**Table S2** Subgroup analyses for factors predicting PFS

| Factor | | Univariate analysis | | Multivariate analysis | |
| --- | --- | --- | --- | --- | --- |
|  |  | HR (95% CI) | *p* value | HR (95% CI) | *p* value |
| Sex | |  |  |  |  |
|  | Male | 1 |  |  |  |
|  | Female | 0.852 (0.410-1.768) | 0.667 |  |  |
| Age | |  |  |  |  |
|  | <60 | 1 |  |  |  |
|  | ≥60 | 0.747 (0.369-1.515) | 0.419 |  |  |
| ECOG PS | |  |  |  |  |
|  | 0 | 1 |  |  |  |
|  | 1 | 1.409 (0.571-3.478) | 0.457 |  |  |
| Location of primary tumor | |  |  |  |  |
|  | Non-rectum | 1 |  |  |  |
|  | Rectum | 0.831 (0.412-1.679) | 0.606 |  |  |
| KRAS | |  |  |  |  |
|  | Wild type | 1 |  |  |  |
|  | Mutant | 0.649 (0.314-1.340) | 0.243 |  |  |
| *UGT1A1* deficient allele | |  |  |  |  |
|  | 0 | 1 |  |  |  |
|  | 1 | 1.579 (0.768-3.245) | 0.214 |  |  |
| Metastatic site, lung | |  |  |  |  |
|  | No | 1 |  |  |  |
|  | Yes | 0.974 (0.417-2.277) | 0.952 |  |  |
| Metastatic site, liver | |  |  |  |  |
|  | No | 1 |  |  |  |
|  | Yes | 1.215 (0.571-2.587) | 0.613 |  |  |
| Metastatic site, peritoneum | |  |  |  |  |
|  | No | 1 |  |  |  |
|  | Yes | 0.948 (0.380-2.363) | 0.909 |  |  |
| Number of metastatic sites | |  |  |  |  |
|  | <3 | 1 |  |  |  |
|  | ≥3 | 0.764 (0.308-1.895) | 0.561 |  |  |
| Irinotecan free interval | |  |  |  |  |
|  | <12months | 1 |  |  |  |
|  | ≥12 months | 0.625 (0.308-1.269) | 0.194 |  |  |

PFS, progression-free survival; HR, hazard ratio; ECOG PS, Eastern Cooperative Oncology Group Performance Status; KRAS Kirsten rat sarcoma

**Table S3** Subgroup analyses for factors predicting overall survival (OS)

| Factor | | Univariate analysis | | Multivariate analysis | |
| --- | --- | --- | --- | --- | --- |
|  |  | HR (95% CI) | *p* value | HR (95% CI) | *p* value |
| Sex | |  |  |  |  |
|  | Male | 1 |  |  |  |
|  | Female | 0.843 (0.397-1.789) | 0.656 |  |  |
| Age | |  |  |  |  |
|  | <60 | 1 |  |  |  |
|  | ≥60 | 0.721 (0.351-1.482) | 0.373 |  |  |
| ECOG PS | |  |  |  |  |
|  | 0 | 1 |  |  |  |
|  | 1 | 2.699 (0.910-8.002) | 0.073 |  |  |
| Location of primary tumor | |  |  |  |  |
|  | Non-rectum | 1 |  |  |  |
|  | Rectum | 1.140 (0.551-2.358) | 0.723 |  |  |
| KRAS | |  |  |  |  |
|  | Wild type | 1 |  |  |  |
|  | Mutant | 1.420 (0.674-2.990) | 0.356 |  |  |
| *UGT1A1* deficient allele | |  |  |  |  |
|  | 0 | 1 |  |  |  |
|  | 1 | 1.211 (0.572-2.562) | 0.617 |  |  |
| Metastatic site, lung | |  |  |  |  |
|  | No | 1 |  |  |  |
|  | Yes | 1.448 (0.582-3.630) | 0.426 |  |  |
| Metastatic site, liver | |  |  |  |  |
|  | No | 1 |  |  |  |
|  | Yes | 1.429 (0.627-3.256) | 0.396 |  |  |
| Metastatic site, peritoneum | |  |  |  |  |
|  | No | 1 |  |  |  |
|  | Yes | 1.470 (0.593-3.647) | 0.406 |  |  |
| Number of metastatic sites | |  |  |  |  |
|  | <3 | 1 |  |  |  |
|  | ≥3 | 1.162 (0.466-2.901) | 0.747 |  |  |
| Irinotecan free interval | |  |  |  |  |
|  | <12months | 1 |  |  |  |
|  | ≥12 months | 0.776 (0.375-1.605) | 0.494 |  |  |

OS, overall survival; HR, hazard ratio; ECOG PS, Eastern Cooperative Oncology Group Performance Status; KRAS Kirsten rat sarcoma

**Table S4** Subgroup analyses for factors predicting overall response

| Factor | | Univariate analysis | | Multivariate analysis | |
| --- | --- | --- | --- | --- | --- |
|  |  | HR (95% CI) | *p* value | HR (95% CI) | *p* value |
| Sex | |  |  |  |  |
|  | Male | 1 |  |  |  |
|  | Female | 0.364 (0.036-3.707) | 0.396 |  |  |
| Age | |  |  |  |  |
|  | <60 | 1 |  |  |  |
|  | ≥60 | 3.714 (0.366-37.708) | 0.267 |  |  |
| ECOG PS | |  |  |  |  |
|  | 0 | 1 |  |  |  |
|  | 1 | 0.261 (0.033-2.089) | 0.206 |  |  |
| Location of primary tumor | |  |  |  |  |
|  | Non-rectum | 1 |  |  |  |
|  | Rectum | 4.308 (0.424-43.733) | 0.217 |  |  |
| KRAS | |  |  |  |  |
|  | Wild type | 1 |  |  |  |
|  | Mutant | 1.393 (0.200-9.711) | 0.738 |  |  |
| *UGT1A1* deficient allele | |  |  |  |  |
|  | 0 | 1 |  |  |  |
|  | 1 | 0.269 (0.027-2.733) | 0.267 |  |  |
| Metastatic site, lung | |  |  |  |  |
|  | No | 1 |  |  |  |
|  | Yes | Not estimable | 0.999 |  |  |
| Metastatic site, liver | |  |  |  |  |
|  | No | 1 |  |  |  |
|  | Yes | 2.000 (0.194-20.614) | 0.560 |  |  |
| Metastatic site, peritoneum | |  |  |  |  |
|  | No | 1 |  |  |  |
|  | Yes | 1.100 (0.100-12.087) | 0.938 |  |  |
| Number of metastatic sites | |  |  |  |  |
|  | <3 | 1 |  |  |  |
|  | ≥3 | 3.833 (0.479-30.700) | 0.206 |  |  |
| Irinotecan free interval | |  |  |  |  |
|  | <12months | 1 |  |  |  |
|  | ≥12 months | 0.776 (0.375-1.605) | 0.494 |  |  |

HR, hazard ratio; ECOG PS, Eastern Cooperative Oncology Group Performance Status; KRAS Kirsten rat sarcoma

**Table S5** Comparison of efficacy and safety outcomes between the UHD trial and later-line clinical trials in metastatic colorectal cancer

| Agents | Regorafenib  vs Placebo | TAS-102 | TAS-102+ Bevacizumab  vs TAS-102 | Fruquintinib  vs Placebo | *UGT1A1*-guided high-dose irinotecan rechallenge |
| --- | --- | --- | --- | --- | --- |
| Trial | CORRECT | RECOURSE | SUNLIGHT | FRESCO-2 | UHD |
| Sample size | 505 vs 255 | 534 vs 266 | 246 vs 246 | 461 vs 230 | 32 |
| Median OS (months) | 6.4 vs 5.0 | 7.1 vs 5.3 | 10.8 vs 7.5 | 7.4 vs 4.8 | 9.3 |
| Median PFS (months) | 2.0 vs 1.7 | 2.0 vs 1.7 | 5.6 vs 2.4 | 3.7 vs 1.8 | 2.9 |
| ORR (%) | 1.0 vs 0.4 | 1.6 vs 0.4 | 6.2 vs 1.0 | 2.0 vs 1.0 | 15.6 |
| DCR (%) | 41 vs 15 | 44 vs 16 | 71 vs 30 | 56 vs 16 | 41 |
| Grade ≥3 AEs | 54% | 69% | 72% | 63% | 59% |
| Common Grade ≥3 AEs | Hand-foot skin reaction (17%)  Fatigue (10%)  Diarrhea (7%)  Hypertension (7%) | Neutropenia (38%)  Leukopenia (21%)  Anemia (18%)  Thrombo-cytopenia (5%) | Neutropenia (43%)  Anemia (6%)  Hypertension (6%)  Thrombo-cytopenia (3%) | Hypertension (14%)  Asthenia (5%)  Hand-foot skin reaction (6%) | Neutropenia (28%)  Nausea (15.6%)  Anemia (6.3%)  Anorexia (6.3%) |

OS, overall survival; PFS, progression-free survival; ORR, objective response rate; DCR, disease control rate; AEs, adverse events;

**Fig. S1** Overall survival by *UGT1A1* allele type

**Figure S2.** Progression-free survival by *UGT1A1* allele type
